# Serum metabolomic signatures of patients with rare neurogenetic diseases: an insight into potential biomarkers and treatment targets

**DOI:** 10.3389/fnmol.2024.1482999

**Published:** 2025-01-10

**Authors:** Nalaka Wijekoon, Lakmal Gonawala, Pyara Ratnayake, Darshana Sirisena, Harsha Gunasekara, Athula Dissanayake, Dhammika Amaratunga, Harry W. M. Steinbusch, Yetrib Hathout, Eric P. Hoffman, Ashwin Dalal, Chandra Mohan, K. Ranil D. de Silva

**Affiliations:** ^1^Interdisciplinary Centre for Innovations in Biotechnology and Neuroscience, Faculty of Medical Sciences, University of Sri Jayewardenepura, Nugegoda, Sri Lanka; ^2^Department of Cellular and Translational Neuroscience, School for Mental Health and Neuroscience, Faculty of Health, Medicine and Life Sciences, Maastricht University, Maastricht, Netherlands; ^3^Lady Ridgeway Hospital for Children, Colombo, Sri Lanka; ^4^Colombo North Teaching Hospital, Ragama, Sri Lanka; ^5^Sri Jayewardenepura General Hospital, Colombo, Sri Lanka; ^6^Teaching Hospital Karapitiya, Galle, Sri Lanka; ^7^Princeton Data Analytics, Princeton, NJ, United States; ^8^School of Pharmacy and Pharmaceutical Sciences, Binghamton University, Binghamton, NY, United States; ^9^Diagnostics Division, Center for DNA Fingerprinting and Diagnostics, Hyderabad, India; ^10^Department of Bioengineering, University of Houston, Houston, TX, United States

**Keywords:** muscular dystrophy, ataxia, biomarker, DMD, LGMD, SCA, pathways

## Abstract

**Introduction:**

To further advance our understanding of Muscular Dystrophies (MDs) and Spinocerebellar Ataxias (SCAs), it is necessary to identify the biological patterns associated with disease pathology. Although progress has been made in the fields of genetics and transcriptomics, there is a need for proteomics and metabolomics studies. The present study aimed to be the first to document serum metabolic signatures of MDs (DMD, BMD, and LGMD 2A) SCAs (SCA 1-3), from a South Asian perspective.

**Methods:**

A total of 28 patients (SCA 1-10, SCA 2-2, SCA 3-2, DMD-10, BMD-2, LGMD-2) and eight controls (aged 8–65 years) were included. Metabolomic analysis was performed by Ultrahigh Performance Liquid Chromatography-Tandem Mass Spectroscopy (UPLC-MS/MS), with support from the Houston Omics Collaborative.

**Results and discussion:**

Amino acid metabolism was the primary altered super pathway in DMD followed by carbohydrate metabolism and lipid metabolism. In contrast, BMD and LGMD 2A exhibited a more prominent alteration in lipid metabolism followed by amino acid metabolism. In SCAs, primarily lipid, amino acid, peptide, nucleotide, and xenobiotics pathways are affected. Our findings offer new insights into the variance of metabolite levels in MD and SCA, with substantial implications for pathology, drug development, therapeutic targets and clinical management. Intriguingly, this study identified two novel metabolites associated with SCA. This pilot cross-sectional study warrants further research involving larger groups of participants, to validate our findings.

## 1 Introduction

Inherited muscular dystrophies (MDs) encompass a range of conditions, including Duchenne muscular dystrophy (DMD), Becker muscular dystrophy (BMD), and limb girdle muscular dystrophy (LGMDs), which arise from mutations in multiple genes (Mercuri and Muntoni, 2013). These diseases are characterized by a gradual decline in muscle tissue and function (Spitali et al., 2018). DMD (OMIM #310200) is the most common type of muscular dystrophy in children, with an incidence rate of 4.80 per 100,000 (Salari et al., 2022). It is characterized by increasing muscle degeneration and weakness, ultimately leading to respiratory failure and premature death (Starosta and Konieczny, 2021). Despite the prevailing knowledge regarding the manifestation of muscular dystrophies, which involves compromised membrane integrity, abnormal calcium homeostasis, chronic inflammation, fibrosis, and impaired tissue remodeling (Deconinck and Dan, 2007), recent studies have revealed metabolic deficits in dystrophic skeletal muscle. These deficits may contribute to the progression of the disease (Xu et al., 2023; Timpani et al., 2015).

Spinocerebellar ataxia (SCA) is a disease that is inherited, progressive, neurodegenerative, and heterogeneous in nature. It primarily affects the cerebellum. SCA is a form of hereditary cerebellar ataxia and is a rare disease (Soong and Morrison, 2018). SCAs occur most often in adults and symptoms include slurred speech, loss of coordination, and balance. Mutations in SCA genes can affect the spinal cord and brainstem cells, and cause damage to cerebellar Purkinje neurons, which results in cerebellar atrophy (Klockgether et al., 2019).

To further advance our understanding of Muscular Dystrophies and Spinocerebellar Ataxias, it is necessary to identify the biological patterns/biomarkers associated with disease pathology (Schumacher-Schuh et al., 2022). Although progress has been made in the fields of genetics and transcriptomics (Lake et al., 2021), there is a need for proteomics and metabolomics studies (Schumacher-Schuh et al., 2022). The most promising biomarkers are those that are responsive to potential disease-modifying therapies and precisely reflect neuropatho- logical and clinical progression (Beckonert et al., 2007). Peripheral biofluid biomarkers, for example serum, have drawn more interest due to their presence in the body (Dunn et al., 2005). Moreover, Blood-based biomarkers offer several benefits, including affordability, user-friendliness, safety, and minimal invasiveness (Gréen, 1969).

Exploring the intricate metabolic alterations associated with MDs and SCAs, metabolomics is essential in developing tailored therapeutic strategies. This thorough understanding paves the way for targeted and personalized approaches, which could improve treatment efficacy and optimize overall care for individuals affected by these disorders, pinpointing metabolic changes associated with the disease (Couch, 2023; Dusek et al., 2023; Gharaba et al., 2023; Khaboushan et al., 2023; Kouba et al., 2023; Morena et al., 2023; Murueta-Goyena et al., 2023; Schultz et al., 2023; Wang et al., 2023; Zhang et al., 2023). Fields such as genomics, proteomics, and transcriptomics assist in elucidating past occurrences and forecasting potential developments in these disorders in the patients. Conversely, metabolomics provide a continuous and current representation of the disease’s status. Consequently, among the diverse omics methodologies, metabolomics currently presents the most promising potential for biomarker identification (Robinson et al., 2014; German et al., 2005). Researchers have demonstrated that metabolomics is promising for identifying neuroactive small-molecule metabolites linked to gut microbiota and the brain possibly through “Gut-brain axis” (Martin et al., 2018), information that other omic methods could not uncover.

A study conducted by Srivastava et al. (2016) examined the metabolites in the serum of patients with multiple forms of muscular dystrophy, including DMD, BMD, LGMD 2B, facioscapulohumeral muscular dystrophy (FSHD), and myotonic dystrophy (DM), in the North and North-East Indian populations. They found that serum from patients with DMD, BMD, FSHD, and DM had elevated levels of branched-chain amino acids (valine, leucine, isoleucine), whereas patients with DMD, BMD, LGMD-2B, and FSHD had reduced levels of glutamine. In addition, the tyrosine levels in the serum of patients with BMD were higher compared with that of normal subjects (Srivastava et al., 2016).

Rybalka et al. (2015) demonstrated that lipid metabolism may be an important metabolic disruption in DMD. In addition, biopsies from individuals with DMD exhibited a reduction in seven metabolites, such as adenosine triphosphate and glycerophosphate choline, and an elevation in 27 metabolites, including sphingomyelin, phosphatidylcholine, phosphatidic acid, and phosphatidylserine (Dabaj et al., 2021; Lindsay et al., 2018). Moreover, Boca et al. (2016) found significant changes in arginine, creatine, creatinine, and androgen derivatives among patients diagnosed with DMD. It is noteworthy that MDs have received limited attention in terms of circulating metabolites and metabolic pathways. Although significant advancements have been achieved, the fundamental pathogenic process of MD requires more study to identify new treatment targets (Xu et al., 2023).

Iorio et al. (1993) examined the serum fatty acid profile using gas chromatographic analysis in patients with Friedreich’s ataxia and SCA, but found no significant differences in the fatty acid profiles of these patients. Griffin et al. (2004) defined a metabolomic phenotype of an SCA3 mouse model using 1H-NMR, which included an increase in glutamine and a decrease in myo-inositol concentration in the brain. Symptomatic SCA3 patients exhibited distinct metabolic profiles with perturbed amino acid and fatty acid metabolism, whereas FFA 16:1, FFA 18:3, L-proline, and L-tryptophan were identified as potential disease biomarkers (Yang et al., 2019); however, metabolomic analysis has not yet been reported in SCA type 1 and 2 patients.

The authors of this study have already provided a detailed description of the proteomic signature of DMD in Sri Lanka, a South Asian population, and proposed an astrocyte-centric pathology for DMD (Wijekoon et al., 2023). In addition, they conducted a proteomic analysis on SCA (Unpublished data). In this study, we examined the biochemical pathways and potential serum metabolomic biomarkers in genetically confirmed patients with muscular dystrophy (DMD, BMD, LGMD 2A) and spinocerebellar ataxia type 1, 2, 3 compared with healthy controls from South Asia. To our knowledge, this is the first study to establish metabolic signatures in the serum of patients with neuromuscular and neurodegenerative diseases.

## 2 Materials and methods

### 2.1 Patient recruitment

This study is a follow-up to a prior study conducted by the principal investigator on individuals with genetically confirmed MD and SCA (Gonawala et al., 2024). Patients were clinically diagnosed by Consultant Neurologist/Consultant Paediatric Neurologist. The sociodemographic characteristics and clinical data of the MD patients were documented using a standard questionnaire and clinical batteries that included the North Star Ambulatory Assessment (NSAA), Vignos scale and Brooke scale. The SCA patients were evaluated using the Scale for the Assessment and Rating of Ataxia (SARA), UHDRS functional score and cognitive assessment using Addenbrooke’s cognitive examination- revised (ACE-r). The participants in this metabolomic study provided their consent, with formal informed consent obtained from each participant where appropriate. In cases where patients were unable to provide consent independently, consent was obtained through a proxy. A total of 28 patients (SCA 1-10, SCA 2-2, SCA 3-2, DMD-10, BMD-2, LGMD-2) and eight controls (aged 8–65 years) were included. Of note, all patients with muscular dystrophy had discontinued corticosteroids for an average of 24 months. This study meets the ethical guidelines of the Sri Lankan institutional review boards which follow the Helsinki Declaration (Ethical Approval Nos. 449/09, 38/19 and 34/14 from The Ethics Review Committee, Faculty of Medical Sciences, University of Sri Jayewardenepura and Ethical Approval No. LRH/D/06/2007 Lady Ridgeway Hospital for Children, Sri Lanka).

A volume of 10 mL of whole blood was collected to vacuum blood collection tubes without anticoagulants (red cap) during blood collection. All the participants in this study did not take any medications or any irritation causing drink/food 72 h before the test. Serum was separated by centrifugation at 3,500 rpm for 20 min. Samples were aliquoted and stored at −80°C until use.

### 2.2 Serum metabolomic analysis

Metabolomic studies were carried out at Metabolon^1^ with support and collaboration from the Houston Omics Collaborative,^2^ with institutional IRB approval obtained from the University of Houston (Houston, TX). The samples were accessioned into the Metabolon laboratory information management system (LIMS) and assigned by LIMS with a unique identifier that was associated with only the original source identifier.

Samples were prepared using the automated MicroLab STAR^®^ system from the Hamilton Company. To remove protein, dissociate small molecules bound to protein or trapped in the precipitated protein matrix, and to recover chemically diverse metabolites, the proteins were precipitated with methanol by vigorously shaking for 2 min (Glen Mills GenoGrinder 2000) followed by centrifugation. The resulting extract was divided into five fractions: two for analysis using two separate reverse-phase (RP)/UPLC-MS/MS methods with positive ion mode electrospray ionization (ESI), one by RP/UPLC-MS/MS with negative ion mode ESI, one by HILIC/UPLC-MS/MS with negative ion mode ESI, and one sample was reserved as a backup. Samples were placed briefly on a TurboVap^®^ (Zymark) to remove the organic solvent. The extracts were stored overnight under nitrogen before preparation for analysis.

### 2.3 Ultrahigh performance liquid chromatography-tandem mass spectroscopy

All methods used a Waters ACQUITY ultra-performance liquid chromatography (UPLC) and a Thermo Scientific Q-Exactive high resolution/accurate mass spectrometer coupled to a heated electrospray ionization (HESI-II) source and an Orbitrap mass analyzer operated at a 35,000 mass resolution. The sample extract was dried, followed by reconstitution in solvents compatible with each of the four methods. Each reconstituted solvent contained a series of standards at fixed concentrations to ensure injection and chromatographic consistency. One aliquot was analyzed using acidic positive ion conditions, which were chromatographically optimized for more hydrophilic compounds. For this method, the extract was gradient-eluted from a C18 column (Waters UPLC BEH C18-2.1 × 100 mm, 1.7 μm) using water and methanol, containing 0.05% perfluoropentanoic acid (PFPA) and 0.1% formic acid (FA). Another aliquot was analyzed under acidic positive ion conditions; however, it was chromatographically optimized for more hydrophobic compounds. In this method, the extract was gradient-eluted from the aforementioned C18 column using methanol, acetonitrile, water, 0.05% PFPA, and 0.01% FA and was operated at an overall higher organic content. Another aliquot was analyzed under basic negative ion optimized conditions using a separate dedicated C18 column. The basic extracts were gradient-eluted from the column using methanol and water containing 6.5 mM ammonium bicarbonate, pH 8. The fourth aliquot was analyzed by negative ionization following elution from an HILIC column (Waters UPLC BEH Amide 2.1 × 150 mm, 1.7 μm) using a gradient consisting of water and acetonitrile containing 10 mM ammonium formate, pH 10.8. MS analysis alternated between MS and data-dependent MSn scans using dynamic exclusion. The scan range varied slightly between methods, but covered 70–1,000 m/z. Raw data was extracted, peak-identified, and QC-processed using Metabolon’s hardware and software.

### 2.4 Statistical analysis

Statistical analyzes were performed in ArrayStudio on log-transformed data. For the analyzes not standard in ArrayStudio, the programs R^3^ or JMP were used. Significance tests (Welch’s two-sample *t*-test), Random forest, and principal components analyzes were used to evaluate the predictive potential of the candidate diagnostic biomarkers.

Random forest analysis was performed to determine which variables (biochemicals) make the largest contribution to the classification; a “variable importance” measure is computed. We used the “Mean Decrease Accuracy” (MDA) as this metric. The MDA was determined by randomly permuting a variable, running the observed values through the trees, and then reassessing the prediction accuracy.

The first principal component was computed by determining the coefficients of the metabolites that maximized the variance of the linear combination. The second component found the coefficients that maximized the variance with the condition that the second component was orthogonal to the first. The third component was orthogonal to the first two components and so on. The total variance was defined as the sum of the variances of the predicted values of each component, and for each component, the proportion of the total variance was computed.

## 3 Results

A total of 28 patients (Male-17, Female-11) exhibiting characteristic clinical findings and genetically confirmed for SCA, DMD, BMD and LGMD (SCA 1-10, SCA 2-2, SCA 3-2, DMD-10, BMD-2, LGMD-2), were included in the study. Table 1 is a summary of the demographic characteristics, genetic mutation and clinical characteristics of the patients.

**Table 1:** Clinical characteristics of the cohort.

Patient ID	Clinical diagnosis	Molecular diagnosis	Sex	Age	Age at onset	Scores for clinical batteries at serum collection
Muscular dystrophy patients (DMD, BMD, and LGMD)						
DMD 01	DMD	*DMD* Duplication-Exon 52–67	Male	7 years	3 years	NSAA-24/34 Vignos Scale-2/9 Brooke Scale-1/6
DMD 02^a^	DMD	*DMD* Deletion-Exon 1–42	Male	11 years	5 years	NSAA-Wheel chair bound Vignos Scale-9/9 Brooke Scale-2/6
DMD 03^a^	DMD	*DMD* Deletion-Exon 1–42	Male	16 years	5 years	NSAA-Wheel chair bound Vignos Scale-9/9 Brooke Scale-2/6
DMD 04	DMD	*DMD* Deletion-Exon 51–55	Male	7 years	3 years	NSAA-21/34 Vignos Scale-2/9 Brooke Scale-1/6
DMD 05	DMD	*DMD* Duplication-Exon 8, 9, and 11	Male	9 years	5 years	NSAA-7/34 Vignos Scale-6/9 Brooke Scale-1/6
DMD 06	DMD	*DMD* Deletion-Exon 51	Male	9 years	4 years	NSAA-17/34 Vignos Scale-3/9 Brooke Scale-1/6
DMD 07^b^	DMD	*DMD* Deletion-Exon 61–62	Male	13 years	5 years	NSAA-Wheel chair bound Vignos Scale-9/9 Brooke Scale-6/6
DMD 08^b^	DMD	*DMD* Deletion-Exon 61–62	Male	13 years	5 years	NSAA-Wheel chair bound Vignos Scale-9/9 Brooke Scale-6/6
DMD 09^c^	DMD	*DMD* Deletion-Exon 44	Male	5 years	4 years	NSAA-N/A Vignos Scale-3/9 Brooke Scale-2/6
DMD 10^c^	DMD	*DMD* Deletion-Exon 44	Male	5 years	4 years	NSAA-N/A Vignos Scale-3/9 Brooke Scale-2/6
BMD 01	BMD	*DMD* Deletion-Exon 45–47	Male	28 years	15 years	NSAA-24/34 Vignos Scale-2/9 Brooke Scale-1/6
BMD 02	BMD	*DMD* Deletion-Exon 45–49	Male	22 years	13 years	NSAA-26/34 Vignos Scale-2/9 Brooke Scale-1/6
LGMD 01^d^	LGMD	*CAPN3* Exon 10 c.1342C > T (LGMD 2A)	Male	23 years	13 years	NSAA-16/34 Vignos Scale-3/9 Brooke Scale-3/6
LGMD 02^d^	LGMD	*CAPN3* Exon 10 c.1342C > T (LGMD 2A)	Female	22 years	15 years	NSAA-15/34 Vignos Scale-4/9 Brooke Scale-3/6
Spinocerebellar ataxia patients (SCA1, SCA2, and SCA3)						
SCA1-01	SCA	CAG expansion-53	Female	38	35	SARA-13 ACE-r-54 UHDRS functional-8
SCA1-02	SCA	CAG expansion-48	Male	53	40	SARA-6.5 ACE-r-31 UHDRS functional-15
SCA1-03	SCA	CAG expansion-52	Female	28	26	SARA-16 ACE-r-65 UHDRS functional-09
SCA1-04	SCA	CAG expansion-63	Female	31	25	SARA-35 ACE-r-10 UHDRS functional-1
SCA1-05	SCA	CAG expansion-61	Female	41	36	SARA-6.5 ACE-r-57 UHDRS functional-22
SCA1-06	SCA	CAG expansion-53	Female	39	32	SARA-25 ACE-r-59 UHDRS functional-03
SCA1-07	SCA	CAG expansion-63	Male	28	27	SARA-13 ACE-r-74 UHDRS functional-16
SCA1-08	SCA	CAG expansion-59	Male	40	30	SARA-8.5 ACE-r-67 UHDRS functional-21
SCA1-09	SCA	CAG expansion-48	Male	40	38	SARA-26 ACE-r-84 UHDRS functional-07
SCA1-10	SCA	CAG expansion-56	Female	47	46	SARA-11 ACE-r-93 UHDRS functional-21
SCA2-01	SCA	CAG expansion-45	Female	45	43	SARA-09 ACE-r-73 UHDRS functional-24
SCA2-02	SCA	CAG expansion-47	Female	39	38	SARA-15 ACE-r-56 UHDRS functional-23
SCA3-01	SCA	CAG expansion-52	Female	38	32	SARA-07 ACE-r-65 UHDRS functional-19
SCA3-02	SCA	CAG expansion-72	Female	41	43	SARA-14 ACE-r-45 UHDRS functional-05

a Siblings of same family.

b Identical twins (zygosity confirmed) with DMD.

c Twins (zygosity not confirmed) with DMD.

d Siblings of same family with three-generation consanguineous relationship.

**Figure 1:**
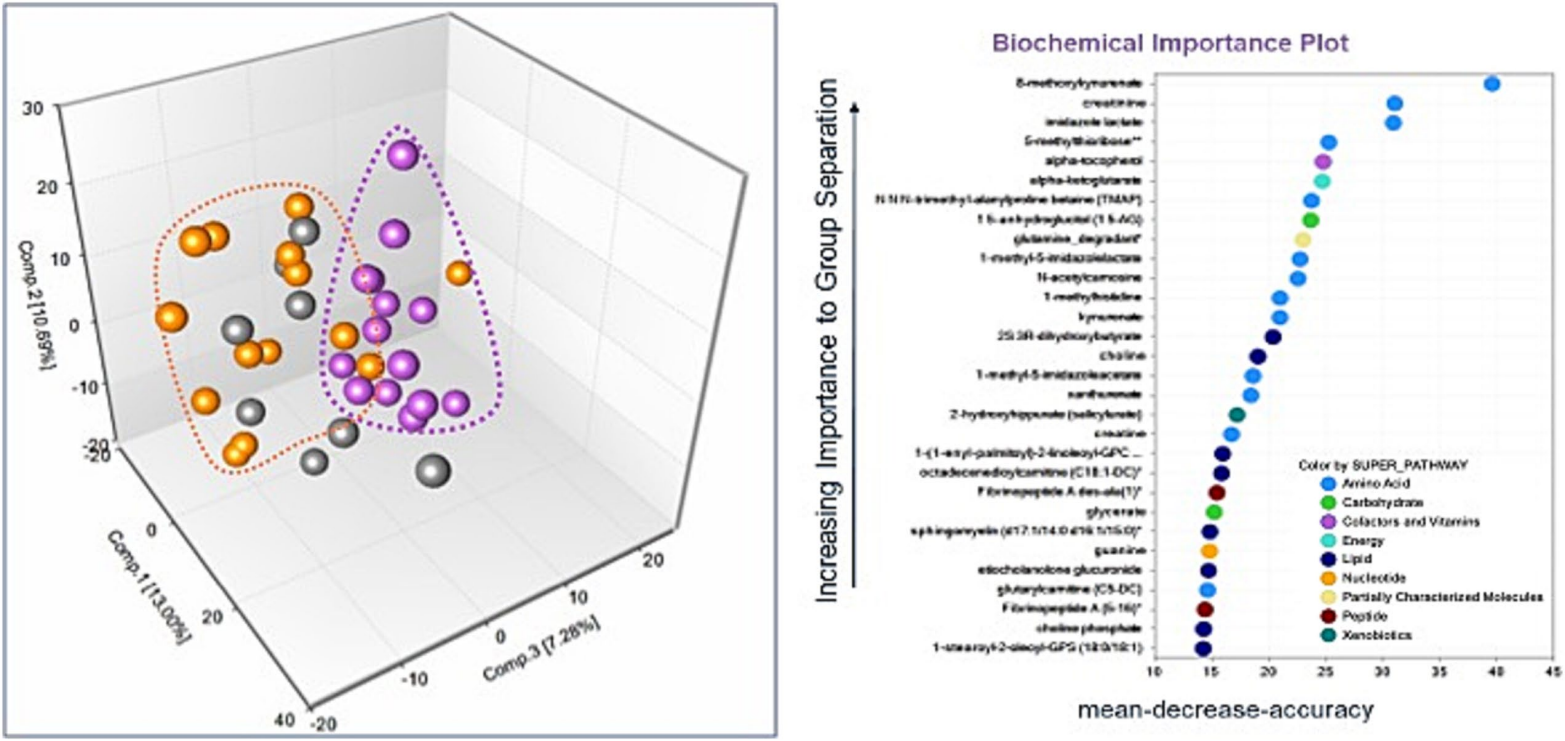
**(A)** The principal components analysis of the SCA samples (purple dots) resulted in their segregation and clustering, with some instances of overlapping with the MD samples (orange dots). The controls are represented by gray-colored dots. **(B)** Random Forest analysis for the identified metabolites. The majority of top 30 biochemicals that aid in group classification were within the amino acid (*n* = 13) and lipid (*n* = 8) metabolism super pathways.

There were 964 known biochemicals detected in this dataset. In a comparison of MD/Control, SCA/Control, and SCA/MD there were 136 (14%), 126 (13%), and 212 (22%) statistically different metabolites, respectively. These results suggest that the metabolomic profiles for the MD and SCA serum samples were significantly different compared with the healthy control serum samples. Principal Component Analysis revealed that the MD samples were segregated and clustered together with some overlapping with the SCA samples (Figure 1A). Similarly, the SCA samples were also segregated and clustered together with some overlapping with the MD samples (Figure 1A). These profiles indicate distinctive metabolomic profiles for both the MD and SCA samples, with some overlapping metabolic signatures.

The metabolomic profiles from a random subset of half of the samples were used to generate a decision tree that was tested on the remaining samples to determine how well they could predict the groupings, with the process repeated thousands of times. There was a high predictive ability of 89% compared with 33% expected by chance. The majority of the top 30 biochemicals that contributed to group classification were associated with the amino acid (Dunn et al., 2005) and lipid (Soong and Morrison, 2018) metabolism super pathways (Figure 1B).

**Figure 2:**
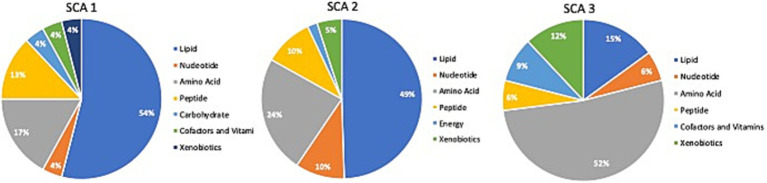
Identification of altered metabolic pathways for SCA (*p* < 0.01). Lipid metabolism was the primary altered super pathway in SCA 1 and SCA 2, which accounted for 54 and 49%, respectively, of the affected metabolic pathways. In contrast, SCA 3 exhibited a more prominent alteration in amino acid metabolism, affecting 52% of the affected metabolic pathways in SCA 3.

**Figure 3:**
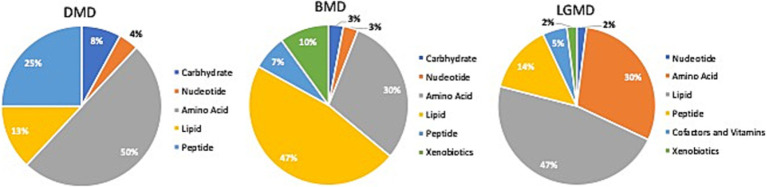
Identification of altered metabolic pathways for MD (*p* < 0.01). Amino acid metabolism was the primary altered super pathway in DMD, which accounted for 50% of the affected metabolic pathways. This was followed by carbohydrate metabolism at 25% and lipid metabolism at 13%. In contrast, BMD and LGMD 2A exhibited a more prominent alteration in lipid metabolism, affecting 47% of the affected metabolic pathways in both BMD and LGMD 2A. Amino acid metabolism accounted for 30% of the affected metabolic pathways in both BMD and LGMD 2A.

Comparison of global biochemical profiles for serum samples from the three different groups (MD, SCA, and control) revealed several key metabolic differences among each disease group shown in Figures 2, 3. In SCAs, primarily lipid, amino acid, peptide, nucleotide, and xenobiotics pathways are affected, whereas in MDs, lipid, amino acid, peptide, and nucleotide pathways are affected. However, from the detected 964 metabolites, 34 (Table 2) were selected, which were highly significant (*p* < 0.01).

**Table 2:** Metabolites identified with significant potential as biomarkers (*p* < 0.001).

Super pathway	Sub pathway	Metabolite	Disease	Upregulated/downregulated
Lipid	Phosphatidylinositol (PI)	1-palmitoyl-2-arachidonoyl-GPI (16:0/20:4)	SCA 2	↑
	Fatty acid, monohydroxy	2-hydroxypalmitate	SCA 2	↑
	Lysophospholipid	1-stearoyl-GPI (18:0)	BMD	↓
	Phospholipid metabolism	Glycerophosphoserine	BMD	↑
	Plasmalogen	1-(1-enyl-palmitoyl)-2-linoleoyl-GPC (P-16:0/18:2)	LGMD2A	↓
	Pregnenolone steroids	Pregnenediol disulfate (C21H34O8S2)	LGMD2A	↑
	Pregnenolone steroids	Pregnenetriol disulfate	LGMD2A	↑
Amino acids	Tryptophan metabolism	Kynurenate	SCA 1	↓
	Glutamate metabolism	N-acetyl-aspartyl-glutamate-(NAAG)	SCA 2	↓
	Polyamine metabolism	N-acetyl-isoputreanine	SCA 2	↓
	Tryptophan metabolism	8-methoxykynurenate	SCA 3	↓
	Leucine, isoleucine and valine metabolism	Valine	SCA 3	↓
	Leucine, isoleucine and valine metabolism	1-carboxyethylvaline	BMD	↑
	Leucine, isoleucine and valine metabolism	3-hydroxy-2-ethylpropionate	BMD	↓
	Glutathione metabolism	5-oxoproline	BMD	↑
	Methionine, cysteine, SAM, and taurine metabolism	Taurine	BMD	↑
	Tyrosine metabolism	3-(4-hydroxyphenyl) lactate	DMD	↓
	Tryptophan metabolism	6-Bromotryptophan	DMD	↑
	Glutamate metabolism	Alpha-ketoglutarate	DMD	↑
	Histidine metabolism	Imidazole lactate	DMD	↓
	Urea cycle, arginine and proline metabolism	N,N,N-trimethyl-alanylproline betaine (TMAP)	DMD	↓
	Tryptophan metabolism	Xanthurenate	DMD	↓
	Acetylasparagine	Citrulline	LGMD2A	↓
	Creatine metabolism	Creatinine	LGMD2A	↓
	Tryptophan metabolism	Kynurenate	LGMD2A	↓
	Tyrosine metabolism	Vanillactate	LGMD2A	↓
Nucleotide	Pyrimidine metabolism	Pseudouridine	SCA 3	↓
Carbohydrate	Glycolysis, gluconeogenesis, and pyruvate metabolism	1,5-anhydroglucitol (1,5-AG)	DMD	↑
	Glycolysis, gluconeogenesis, and pyruvate metabolism	Lactate	BMD	↑
Peptide	Gamma-glutamyl amino acid	Gamma-glutamyl-leucine	DMD	↓
	Gamma-glutamyl amino acid	Glutamine degradant	DMD	↓
	Gamma-glutamyl amino acid	Gamma-glutamyltyrosine	LGMD2A	↓
	Fibrinogen cleavage peptide	Fibrinopeptide B (1–11)	LGMD2A	↑

**Figure 4:**
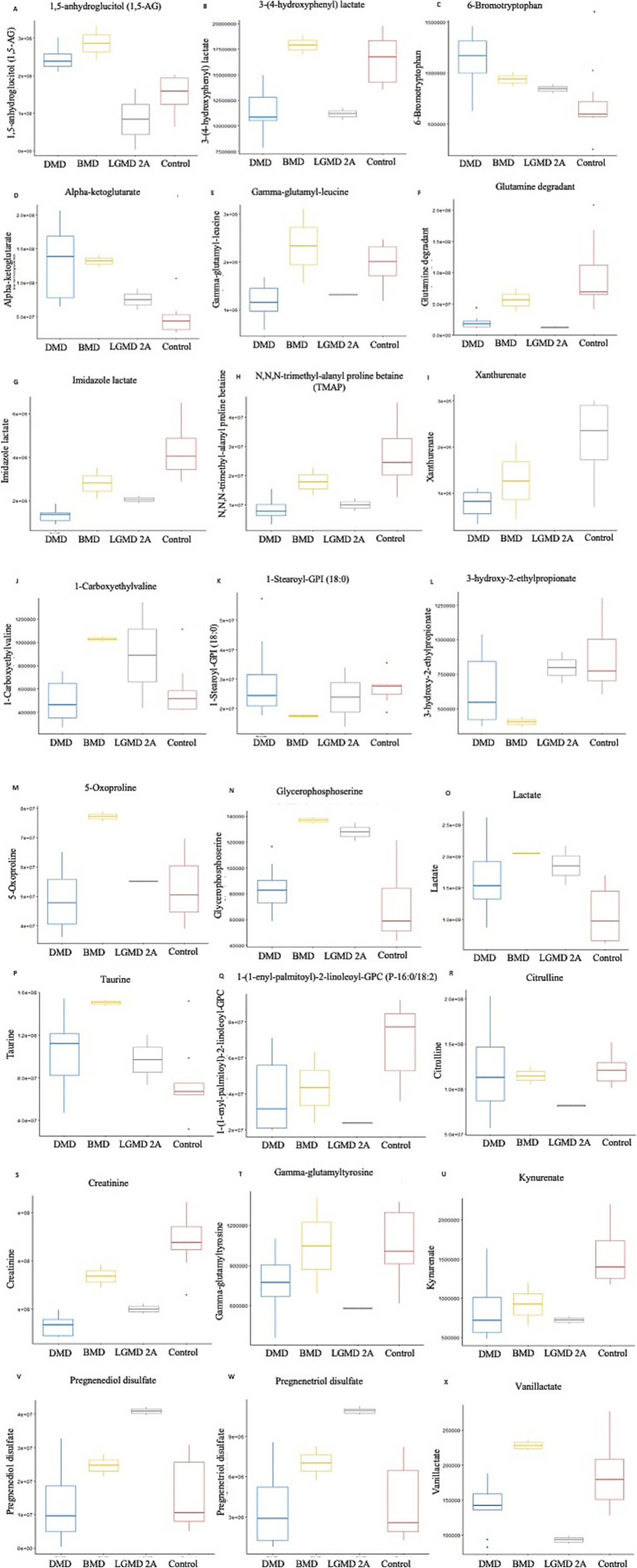
Metabolites identified with significant potential as biomarkers (*p* < 0.001) in Muscular Dystrophies. **(A–H)** Shows the highly significant (*p* < 0.001) metabolites in DMD, **(I–P)** represents the highly significant (*p* < 0.001) metabolites in BMD, and **(Q–X)** represents the highly significant (*p* < 0.001) metabolites in LGMD 2A.

In the present study, even though metabolites related to carbohydrate metabolism (Table 2; Figures 3, 4A) and peptide metabolism (Table 2; Figures 3, 4E,F) found to be altered in DMD, a comparison of the altered metabolic super pathways of MDs revealed that amino acid metabolism was the primary altered super pathway in DMD, which accounted for 50% of the affected metabolic pathways (Figures 3, 4B–D,G–I; Table 1). In contrast, BMD and LGMD 2A exhibited a more prominent alteration in lipid metabolism, which was associated with 47% (Figure 3) of the affected metabolic pathways in both BMD (Table 2; Figures 4K,N), and LGMD 2A (Table 2; Figures 4Q,V,W). Amino acid metabolism accounted for 30% (Figure 3) of the affected metabolic pathways in both BMD (Table 2; Figures 4J,L,M,P) and LGMD 2A (Table 2; Figures 4R,S,U,X). In addition, carbohydrate metabolism was found to be linked to just 3% of the altered metabolic pathways in BMD (Figures 3, 4; Table 2), but not in LGMD. On the other hand, in LGMD2A, 14% of the altered metabolic super pathways were linked to peptide metabolism (Figures 3, 4T; Table 2), but in BMD, this percentage was only 7% (Figure 3).

Significant differences (*p* < 0.01) were observed in fatty acid metabolism in the serum of individuals with DMD, BMD, and LGMD2A in the present study. Decanoylcarnitine (C10) and deoxycarnitine levels were found to be downregulated in individuals with DMD. In BMD, drenoylcarnitine (C22:4) and dihomo-linolenoylcarnitine (C20:3n3 or 6) were upregulated, whereas cis-3,4-methylene-heptanoylcarnitine and laurylcarnitine (C12) were downregulated. Similarly, in LGMD2A, cis-4-Decenoylcarnitine, decanoylcarnitine (C10), octanoylcarnitine (C8), and octadecenedioylcarnitine (C18:1-DC) levels were decreased.

## 4 Discussion

The present study aimed to be the first to document serum metabolic signatures of MDs (DMD, BMD, and LGMD 2A) and SCAs (SCA 1-3) in a Sri Lankan patient cohort (Table 1), from a South Asian perspective.

### 4.1 Metabolites in MDs

In the present study, we found that in blood samples of individuals with DMD, there was a significant upregulation (*p* < 0.001) of AKG levels (Table 2) and a significant downregulation (*p* < 0.01) of KGM levels. The decrease in KGM expression may be attributed to the activation of the glutaminase II pathway, which results in an increase in AKG levels. This increase in AKG levels may counteract protein degradation and muscle dysfunction in DMD. Cai et al. (2018) showed that *α*-ketoglutarate (AKG) successfully reduced protein degradation and ameliorated muscle atrophy and dysfunction in a DMD mouse model. This suggests that AKG may be used for the treatment of muscle dysfunction in DMD (Cai et al., 2018). Nevertheless, it is important to note that there is an alternative pathway known as the glutaminase II pathway, which is often overlooked in the transformation of glutamine into AKG. In this pathway, the transamination of glutamine results in the production of the equivalent α-keto acid, α-ketoglutaramate (KGM). The hydrolysis of KGM by omega-amidase results in the formation of AKG and ammonia (Cooper and Kuhara, 2014).

Moreover, Buddington et al. (2004) found that AKG, either generated by gut bacteria or obtained from food, can be absorbed by intestinal enterocytes and metabolized, even if it is not used by microbes. Therefore, AKG may be an important dietary supplement for animal and human nutrition, as it has the potential to enhance cellular energy, boost immunity, and promote overall health (Pierzynowski and Pierzynowska, 2022; He et al., 2015). This implies that diets that promote the growth of gut microbiota may also promote the production of AKG, which may be a viable strategy to mitigate protein breakdown and muscle dysfunction in DMD.

Srivastava et al. (2016) analyzed serum using NMR spectroscopy to identify various forms of muscular dystrophy. They found that patients with DMD, BMD, and FSHD have higher levels of branched-chain amino acids (BCA) compared with normal individuals; however, this was not observed in LGMD 2B patients (Srivastava et al., 2016). Our study on DMD, BMD, and LGMD 2A revealed a notable change (*p* < 0.01) in the components of the BCA metabolism pathway in both BMD and LGMD 2A; however, no significant difference was observed in DMD at a *p* < 0.01 level. Nevertheless, at a significance threshold of *p* < 0.05, we observed a significant change in the components of the BCA metabolism pathway in our DMD samples. In BMD, the components involved in the BCA metabolism pathway, including 1-carboxyethylvaline and 3-hydroxy-2-ethylpropionate, were upregulated (*p* < 0.001) and downregulated (*p* < 0.001), respectively, as listed in Table 2 and Figures 4J,L. Based on our data, leucine and isoleucine were downregulated (*p* < 0.01) in LGMD 2A. The concentration of serum BCA is increased in several pathological states, including diabetes mellitus, starvation, obesity, and maple syrup urine disease (Oglesbee et al., 2008; Felig et al., 1977). However, the participants involved in the present study did not exhibit any of these pathological conditions. Thus, elevated BCA in the serum resulting from these clinical conditions can be excluded. Furthermore, an elevated concentration of BAA in the serum is detected as a result of increased activity of cathepsin A, cathepsin B1, and dipeptidyl peptidases in the dystrophic muscle that is impacted (Srivastava et al., 2016; Kar and Pearson, 1977). Moreover, the higher levels of serum BCA in patients with DMD, BMD, and LGMD can be attributed to a reduction in protein synthesis (Rennie et al., 1982).

In the present study a significant differences (*p* < 0.01) were observed in fatty acid metabolism in the serum of individuals with DMD, BMD, and LGMD2A. Of note, dysregulated fatty acid metabolism was shown to contribute to muscle lipid accumulation (Jang et al., 2016); therefore, it is possible that it contributes to the increased muscle fat replacement observed in MD patients, and may represent a therapeutic target (Xu et al., 2023). In addition, we found a decrease in the levels of certain unsaturated fatty acids, specifically cis-3,4-methylene-heptanoylcarnitine and cis-4-decenoylcarnitine, which suggests a disruption in energy metabolism in MD and a potential association with muscle cell necrosis (Signorelli et al., 2023). Because of the important role of fatty acid oxidation in energy metabolism, a decrease in this process could potentially lead to fibrosis resulting from a disruption in energy metabolism. Interestingly, studies have shown a decrease in fatty acid oxidation in individuals with DMD, which may be associated with the development of muscle fibrosis during this condition. This finding offers new opportunities for research on fibrosis in DMD.

Our study revealed dysregulated lipid metabolism. In BMD, the dysregulation of components in the lysophospholipid pathway and phospholipid metabolism pathway was statistically significant (*p* < 0.001) (Figures 4K,N). Interestingly, reports have indicated that alterations in Ca^2+^ homeostasis and phospholipid homeostasis in DMD result in vulnerability of the mitochondrial membrane and alterations of its structure, which in turn, disrupt energy production and oxidative metabolism. Consequently, the disturbance in phospholipid metabolism may be associated with muscular dystrophy (Dubinin et al., 2020; Lin et al., 2019; Hughes et al., 2019; Kuno et al., 2018). Laurila et al. (2022) discovered that blocking the production of sphingolipids effectively combats muscular dystrophy. Thus, hindering the production of sphingolipids may represent an approach to treating muscular dystrophy.

Remarkably, our results indicated a significant downregulation of xanthurenate, and 6-bromotryptophan in DMD, which are involved in tryptophan metabolism. In LGMD, kynurenate and vanillactate was significantly downregulated which are involved in tryptophan metabolism and tyrosine metabolism, respectively (Table 2). Moreover, glycocholate, a molecule involved in primary bile acid metabolism, was increased in DMD along with AAA (*p* < 0.01). Furthermore, taurocholate sulfate, a molecule involved in secondary bile acid metabolism, was decreased in LGMD (*p* < 0.01). It is noteworthy that, microbiome metabolism is characterized by changes in aromatic amino acids (phenylalanine, tyrosine, and tryptophan) and bile acid metabolism. The gut microbiome produces a variety of metabolites through the breakdown of aromatic amino acids (AAA), which may influence immune, metabolic, and neuronal responses in nearby and distant areas (Liu et al., 2020).

In this context, there has been a recent emergence of the “gut-muscle axis” hypothesis, which suggests that a two-way communication exists between the gut microbiota and the muscular system. This communication is believed to regulate muscular function and may be disrupted. Nevertheless, there is limited knowledge regarding the specific molecular mechanisms connecting intestinal microorganisms to skeletal muscle (Jayaraman and Pettersson, 2023). This highlights the importance of gaining a deeper understanding of DMD development and the associations between the affected organs, including crosstalk between the gut microbiota and skeletal muscle. Examining the potential interactions between the gut microbiota and skeletal muscle will provide valuable insight in this regard (Jollet et al., 2024). Therefore, our findings on the involved in tryptophan metabolism and tyrosine metabolism in MDs may provide a foundation for future studies on the involvement of the “gut-muscle axis” in muscular dystrophies, which represents a potential strategy for new MD therapies. Moreover, This concept is reinforced by the findings of Kalkan et al. (2023) who demonstrated a notable difference in the gut microbiota composition between wild-type and mdx mice. In addition, they discovered that the administration of a metabolically supporting anti-inflammatory drug restored the microbiome composition in mdx mice (Kalkan et al., 2023).

### 4.2 Metabolites in SCAs

**Figure 5:**
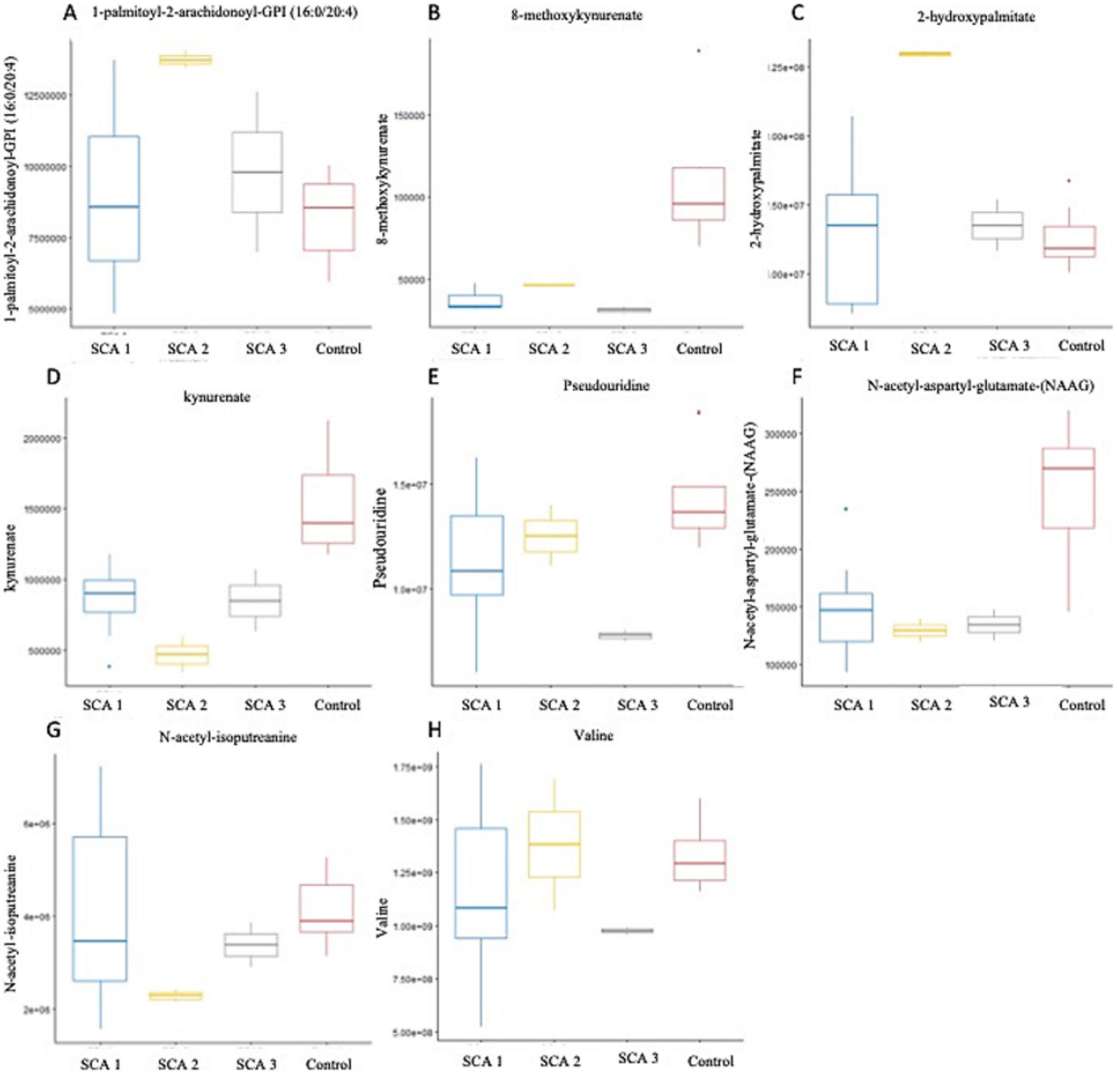
Metabolites identified with significant potential as biomarkers (*p* < 0.001) in Spinocerebellar Ataxia type 1–3. **(A–H)** Represent metabolites significantly (*p* < 0.001) associated with SCAs. In SCA type 1, kynurenate was downregulated; SCA type 2, 1-palmitoyl-2-arachidonoyl-GPI (16:0/20:4), 2-hydroxypalmitate were upregulated. N-acetyl-aspartyl-glutamate (NAAG) and N-acetyl-isoputreanine were downregulated; SCA type 3-8-methoxykynurenate, valine, and pseudouridine were downregulated.

Significant amino acids observed in patients with SCA include kynurenate, 8-methoxykynurenate, N-acetyl-isoputreanine, N-acetyl-aspartyl-glutamate (NAAG), and valine (Figures 5A–H). Tryptophan metabolism was attenuated in patients with SCA 1 and 3 (Table 2). Tryptophan is an important amino acid that plays a central role in protein production. Approximately 1% of the total body amount of tryptophan undergoes conversion into serotonin, whereas the majority, approximately 95%, is metabolized through the kynurenine pathway. This pathway ultimately results in the production of the oxidized form of nicotinamide adenine dinucleotide (NAD+), which is a coenzyme involved in a variety of cellular processes (Mor et al., 2021).

In this study, we observed the considerable downregulation of kynurenate (kynurenic acid) and 8-methoxy-kynurenate, which are metabolites of the kynurenine pathway, in patients with SCA 1 and SCA 3, respectively (Figure 5D). Solvang et al. (2022) conducted a review of the literature and found that individuals with Alzheimer’s disease (AD) have reduced plasma levels of kynurenate. This suggests a transition toward neurotoxic metabolites, rather than neuroprotection, in the peripheral kynurenine pathway associated with these disorders (Solvang et al., 2022). A comparable pattern was observed in individuals with Parkinson’s disease (PD) and Huntington’s disease (HD), as well as children diagnosed with autism spectrum disorders. Conversely, in cases of vascular cognitive dementia, there was an elevation in serum concentration (Boros and Vécsei, 2019). The observation is significant because reduced levels of kynurenate can trigger an insufficient anti-inflammatory reaction, resulting in increased tissue damage and excessive cell growth during inflammation in AD, PD, HD, and Lewy body dementia. This, in turn, exacerbates their symptoms (Mor et al., 2021).

N-acetyl-isoputreanine has an important role in polyamine metabolism and a significant decrease was observed in patients with SCA 2 in the present study (Figure 5G). Shi et al. (2019) found that N-acetyl-isoputreanine is linked to the cognitive domain of processing speed in normal individuals. N-acetyl-isoputreanine is a byproduct of the enzymatic activity of aldehyde dehydrogenase (ALDH) and is produced through polyamine metabolism (Seiler, 1990). Our findings indicated a similar molecular mechanism of action between ALDH activity and polyamine metabolism, which has been linked to cognitive impairment (Shi et al., 2019; Fitzgerald et al., 2017; Grünblatt and Riederer, 2016).

In the present study, the amino acid N-acetyl-aspartyl-glutamate (NAAG), which is involved in glutamate metabolism, was markedly reduced in patients with SCA 2 (Figure 5F). The dipeptide NAAG is highly prevalent in the brain and acts as a neuromodulator of glutamatergic synapses by activating presynaptic metabotropic glutamate receptor 3 (mGluR3). NAAG may play a role in various diseases and conditions, including stroke, traumatic brain injury, epilepsy, age-related neurodegenerative diseases, schizophrenia, and pain, by regulating glutamate release (Neale and Yamamoto, 2020). Previous studies indicate that patients with Alzheimer’s disease (AD) have reduced NAAG levels in the brain regions most affected compared with individuals who are in good condition (Jaarsma et al., 1994). Furthermore, mGluR3 exhibits strong expression in the basal ganglia circuitry of animals (Testa et al., 1994) and humans (Samadi et al., 2009). Of note, mGluR3 is particularly abundant in the caudate nucleus, where its expression is significantly reduced in PD patients and PD models compared with control subjects (Chen and Li, 2019). These results indicate that NAAG and other mGluR3 agonists may serve as valuable neuroprotective therapeutic interventions for individuals with PD (Morland and Nordengen, 2022).

Patients with SCA 3 exhibit notable reductions in valine levels (Figure 5H), which are derived from the metabolism of leucine, isoleucine, and valine sub pathway. A recent study provided evidence of a major disruption in the amino acid metabolism pathway within the symptomatic SCA3 group. This study found a decrease in the expression of branched-chain amino acids, such as valine and leucine, as well as aromatic amino acids, including tryptophan and tyrosine, in the serum of individuals with SCA3, which is consistent with the downregulation of valine observed in the present study. Furthermore, González-Domínguez et al. (2015) found a notable reduction in valine levels in the serum of recently diagnosed sporadic Alzheimer’s disease (AD) patients, who had not been administered any medication. Toledo et al. (2017) conducted a recent study involving a large number of subjects and found a correlation between decreased plasma valine levels and the rate of cognitive decline. Significant changes in the metabolism of tiglylcarnitine (C5:1-DC) and isovalerylglycine, were observed in Fragile X-associated Tremor/Ataxia Syndrome, which are metabolites of the leucine, isoleucine, and valine pathways (Zafarullah et al., 2020).

In the present study, a notable increase in the expression of 1-palmitoyl-2-arachidonoyl-GPI (16:0/20:4) (Figure 5A) and 2-hydroxypalmitate (Figure 5C) was observed within the subpathways of lipid metabolism, specifically, phosphatidylinositol (PI) and fatty acid, monohydroxy, respectively, among individuals diagnosed with SCA 2. The metabolites, 1-palmitoyl-2-arachidonoyl-GPI (16:0/20:4) and 2-hydroxypalmitate, have not been previously reported to be associated with SCAs or other neurodegenerative disorders. Secondary messengers in cells, which regulate numerous processes, such as phagocytosis, migration, and endocytosis, are phosphorylated derivatives of phosphatidylinositol (PI). PI levels, their interactions, and concentration gradients intricately attenuate signaling cascades associated with membrane trafficking, protein turnover, actin remodeling, and clearance of accumulated proteins. The correlation between various gene mutations and neurodegenerative illnesses in the phosphoinositide pathway highlights the need for new therapeutic strategies (Desale and Chinnathambi, 2021).

Yang et al. (2019) recently reported that symptomatic SCA3 patients experienced severe disruptions in fatty acid metabolism. The process of *β*-oxidation of free fatty acids (FFA) involves the breakdown of fatty acids in different tissues to produce energy. They observed a decrease in the serum levels of saturated fatty acid among symptomatic individuals with SCA3, whereas the levels of monounsaturated fatty acid (MUFA) and polyunsaturated fatty acid (PUFA) showed an increase. Specifically, the levels of palmitic acid (FFA 16:0) and stearic acid (FFA 18:0) decreased, whereas palmitoleic acid (FFA 16:1), oleic acid (FFA 18:1), linoleic acid (FFA 18:2), and linolenic acid (FFA 18:3) increased. Thus, these fatty acids have the potential to be used as a new diagnostic method for detecting SCA3 (Yang et al., 2019).

Elevated levels of the nucleotide pseudouridine (Figure 5E) have been observed in patients with SCA 3, indicating its role as a byproduct of pyrimidine metabolism. Pseudouridine is widely distributed in various noncoding RNA molecules and has also been observed in coding mRNA recently (Li et al., 2015; Schwartz et al., 2014; Lovejoy et al., 2014; Carlile et al., 2014). Evidence suggests that pseudouridine may play a role in regulating neuronal function. For example, individuals diagnosed with mild to moderate Alzheimer’s disease (AD) have notably higher concentrations of urinal pseudouridine (Lee et al., 2007); however, the potential association between this increase and the etiology of Alzheimer’s disease remains unclear. Moreover, studies have proposed that pseudouridylation may function as a means to directly detect oxidative stress, a condition that has been associated with a heightened susceptibility to neurodegeneration (Uttara et al., 2009). deLorimier et al. (2017) revealed a clear connection between pseudouridine and neuronal abnormalities in patients with type 2 myotonic dystrophy. They demonstrated that pseudouridine decreases the binding of MBNL1 at extended CCUG repeats, which is responsible for causing illness (Angelova et al., 2018; deLorimier et al., 2017).

**Figure 6:**
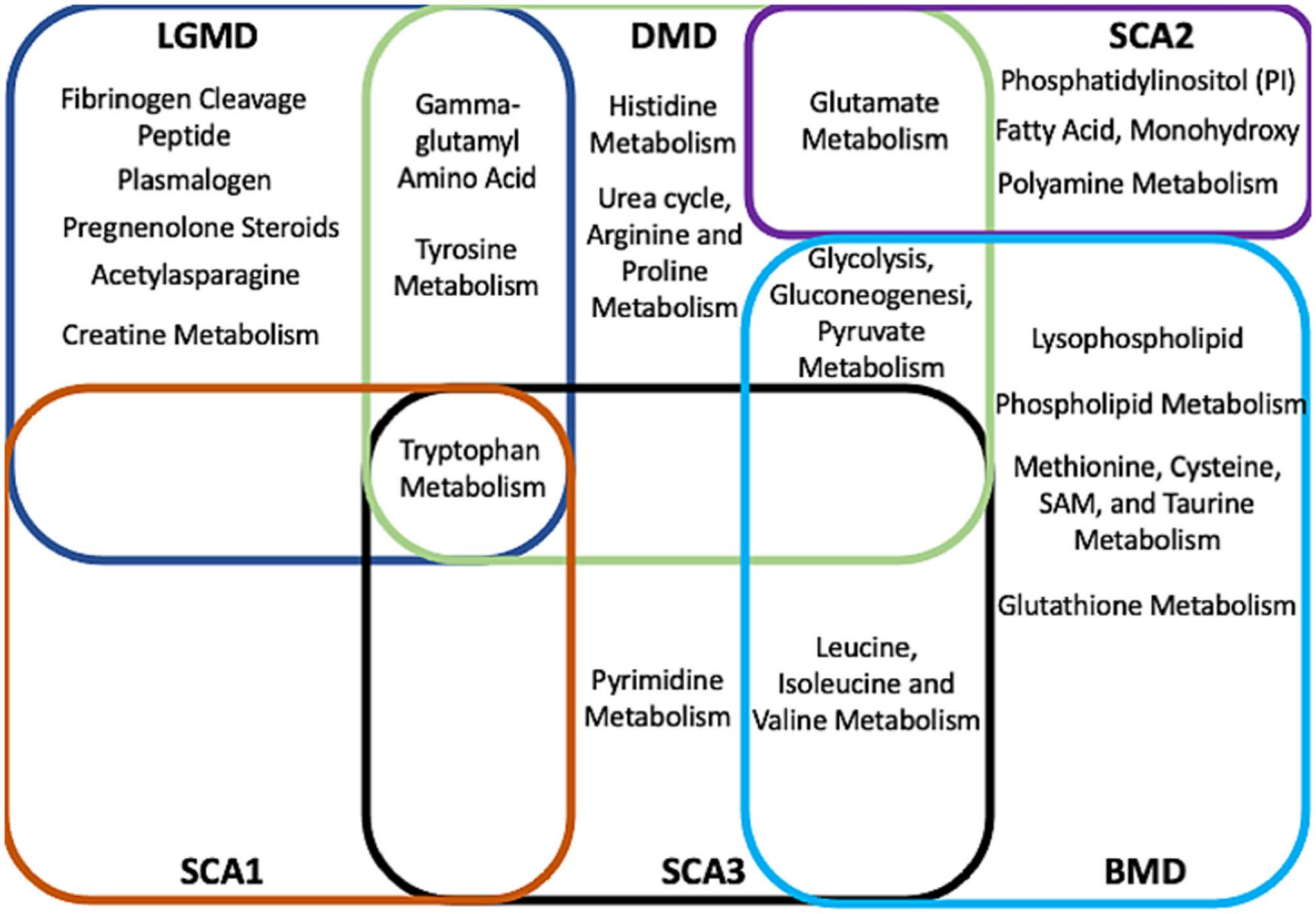
Venn diagram showing a possible shared pathological mechanism in MD and SCA. Red- metabolites of SCA1, Purple- metabolites of SCA2, Black- metabolites of SCA3, Dark blue- metabolites of LGMD, Green- metabolites of DMD, Light blue- metabolites of BMD.

As shown in venn diagram (Figure 6), following metabolic sub-pathways were identified as commonly altered in various forms of MDs and SCAs; Tryptophan metabolism in DMD, LGMD, SCA1, SCA3; Leucine- Valine metabolism in BMD and SCA3; Glutamate metabolism in DMD and SCA2; Glycosis- Pyruate metabolism in DMD and BMD; Gamma- Glut metabolism in DMD and LGMD. This suggests a possible shared pathological mechanism in MD and SCA.

Despite the presentation of a novel research approach for examining the pathophysiology of Muscular Dystrophies and Spinocerebellar Ataxias, there are several limitations to this study. First, the sample size was limited. Further studies using a larger cohort with more comprehensive medication and diet histories are needed to confirm the initial findings and the role of the putative biomarkers. However, it is important to note that this study represents the first exploratory investigation into metabolic alterations in individuals with muscle dystrophies and spinocerebellar ataxias in Sri Lanka. It was also a pilot cross-sectional study, which may only capture transient metabolic disturbances. Nevertheless, the data provides a fundamental basis for subsequent longitudinal investigations. Second, it is necessary to conduct a comprehensive study to evaluate our selected metabolic markers using target validation. It is important to emphasize that the chemical content of metabolites in blood may not accurately reflect the concentration in the brain. In addition, collecting cerebrospinal fluid (CSF) or brain tissue is intrusive and subject to ethical constraints. However, at the time of preparing this publication, one of the patients diagnosed with SCA type 1 who had previously consented to provide her cadaver for future research had unfortunately passed away. Consequently, we are considering a follow-up study that will make use of this cadaver.

## 5 Conclusion

Our findings offer new insights into the variance of metabolite levels in MD and SCA, with substantial implications for pathology, drug development, therapeutic targets and clinical management. In this context, we have identified potential therapeutic targets for SCA 2 and MD. These include phosphatidylinositol and NAAG for SCA 2, and α-ketoglutarate and dysregulated fatty acid metabolism for MD. Intriguingly, this study identified two novel metabolites associated with SCA namely 1-palmitoyl-2-arachidonoyl-GPI (16:0/20:4) and 2-hydroxypalmitate. In addition, the comparative analysis of metabolites revealed that the altered super pathway in DMD, SCA1, SCA2, SCA 3 were primarily related to amino acid metabolism. In contrast, SCA 2, BMD and LGMD 2A showed a significant change in lipid metabolism. This may suggest a possible shared pathological mechanism in MD and SCA. Compounds associated with the gut microbiome were found in both MD and SCA samples. This emphasizes the significance of acquiring a deeper understanding of the development of these diseases and the crosstalk between the gut microbiota. This pilot cross-sectional study warrants further research involving larger groups of participants, to validate our findings and explore the potential of these metabolites as diagnostic methods, biomarkers and therapeutic targets for these diseases.

## Data Availability

The raw data supporting the conclusions of this article will be made available by the authors, without undue reservation.

## Footnotes

^1^ https://www.metabolon.com/about-us/

^2^ https://hoc.bme.uh.edu/

^3^ http://cran.r-project.org/
